# Vibration Sensors on Flexible Substrates Based on Nanoparticle Films Grown by Physical Vapor Deposition

**DOI:** 10.3390/ma17071522

**Published:** 2024-03-27

**Authors:** Evangelos Aslanidis, Savvas Sarigiannidis, Evangelos Skotadis, Dimitris Tsoukalas

**Affiliations:** 1 Department of Applied Physics, National Technical University of Athens, 15780 Athens, Greece; evaaslani@central.ntua.grsansarigiannidis@mail.ntua.gr (S.S.); evskotad@central.ntua.gr (E.S.); 2 Institute of Electronic Structure and Laser, Foundation for Research & Technology Hellas, N.Plastira 100, Voutes, 70013 Heraklion, Greece

**Keywords:** vibration sensor, strain sensor, nanoparticles, physical vapor deposition, platinum, e-skin, acoustics

## Abstract

Flexible electronics have gained a lot of attention in recent years due to their compatibility with soft robotics, artificial arms, and many other applications. Meanwhile, the detection of acoustic frequencies is a very useful tool for applications ranging from voice recognition to machine condition monitoring. In this work, the dynamic response of Pt nanoparticles (Pt NPs)-based strain sensors on flexible substrates is investigated. the nanoparticles were grown in a vacuum by magnetron-sputtering inert-gas condensation. Nanoparticle sensors made on cracked alumina deposited by atomic layer deposition on the flexible substrate and reference nanoparticle sensors, without the alumina layer, were first characterized by their response to strain. The sensors were then characterized by their dynamic response to acoustic frequency vibrations between 20 Hz and 6250 Hz. The results show that alumina sensors outperformed the reference sensors in terms of voltage amplitude. Sensors on the alumina layer could accurately detect frequencies up to 6250 Hz, compared with the reference sensors, which were sensitive to frequencies up to 4250 Hz, while they could distinguish between two neighboring frequencies with a difference of no more than 2 Hz.

## 1. Introduction

The development of high-sensitivity and low-cost strain sensors based on nanomaterials has been a subject of intensive research during the past decade, with applications ranging from electronic skin [1,2] and motion detection [3] for healthcare and wearables [4] to structural health monitoring of large-scale constructions [5]. Along these lines, various nanomaterials have been investigated, including silver nanowires [6], carbon nanotubes [7,8], graphene [9], thin cracked metallic films [10], and metallic nanoparticles [11,12]. 

Metallic nanoparticle (NP) strain sensor operation, in particular, is based on the resistance change of a nanoparticle network that is formed between two metallic electrodes due to changes in the inter-nanoparticle distance. Such changes occur with increasing strain, which affects charge transport between distinct nanoparticles or nanoparticle clusters [13,14]. It is worth noting that in the case of metallic nanoparticle films, charge transport is based on quantum mechanical phenomena (i.e., electron tunneling, variable range hopping), while most devices that fall in this regime showcase a thermally activated Arrhenius-type conductivity. In this case, conductivity is exponentially dependent on inter-nanoparticle distance and activation energy, meaning that nanoparticle-based devices can significantly outperform (in terms of sensitivity) traditional metallic strain gauges [13]. In most cases, the NP material is preferably a noble metal [15] in order to avoid oxidation and, hence, modification of network resistance during the sensor’s lifetime. With respect to the available fabrication methods for metallic nanoparticles or nanoclusters, various techniques have been proposed, including the chemical synthesis of NPs (amongst the most common techniques) [16] which are then deposited on top of a substrate by ink-jet printing, drop-casting, or spin coating, the thermal evaporation of a thin metal film, followed by its annealing [17], the green synthesis of metal (or metal oxide) “biogenic” NPs via sustainable and eco-friendly methods that often employ specific bio-materials (e.g., bacteria, fungi, algae, plant extracts, etc.) [18], and, finally, the formation and deposition of the NP film by gas condensation in a vacuum. 

**Figure 1:**
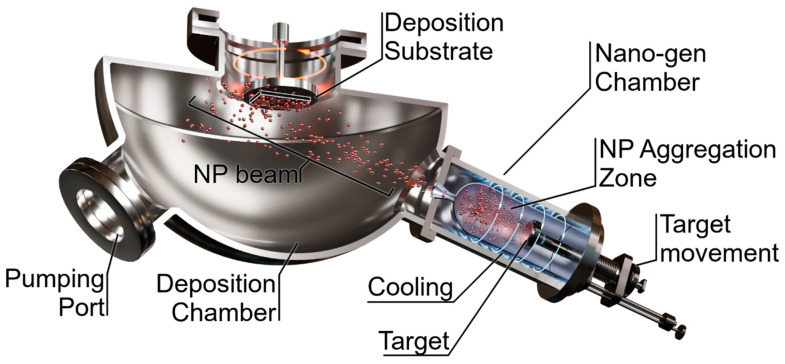
Experimental vacuum setup showing the nanoparticle generation and deposition system based on DC sputtering and gas condensation.

The latter physical vapor deposition (PVD) technique employs either direct current (DC) or radio frequency (RF) sputtering. It is worth noting that, unlike complex chemical synthesis methods, this technique does not involve hazardous chemical reagents. In DC sputtering, ions of an inert gas bombard a metallic target, causing gas-phase atoms from the target’s surface to detach. Due to a pressure difference between the nanoparticle generation chamber and the deposition chamber (Figure 1), the detached atoms condense as they move towards the deposition chamber. This process enables straightforward control over nanoparticle size and facilitates their uniform coverage on top of the deposition substrate. Moreover, the entire process is conducted at room temperature. Metallic nanoparticles can also be produced by sputtering a metal target, followed by thermal treatment of the deposited metallic film; this, in turn, results in material aggregation and, hence, nanoparticle formation. Since the sputtering technique is widely used in the microelectronics industry, it is suitable for mass production and batch fabrication, as well as for research purposes. Numerous publications can be found on sputtering principles, theoretical studies regarding particle formation, and enhancements to sputtering systems to achieve improved properties [19].

Furthermore, it has also been reported [20] that NP strain sensors have the potential to detect acoustic vibrations with an infinitesimal amplitude, which extends the application field of NP arrays towards precision devices with ultrafast dynamics. This possibility opens the way to exploit NP strain sensors as vibration sensors, which are of interest in various application fields ranging from voice recognition [21] to condition-based monitoring (CbM) of machines, which can enable cost savings on machine life and maintenance [22]. A recent review on the use of strain sensors for vibration sensing suggested that resistive strain sensors have a potential that needs to be further investigated, due mainly to their low cost and low weight, especially when compared to accelerometers, which are currently the industry standard for vibration sensing and monitoring [23]. 

It is worth remarking that although the dynamic behavior of NP strain sensors has been reported (refs. [19,20]), this pertains to an array of chemically synthesized nanoparticles which, due to coating of the nanoparticle core with surfactants and ligands, exhibit a different behavior [24] than ligand-free NPs. Thus, a study regarding the dynamic response of a ligand-free physically synthesized nanoparticle strain sensor is missing in the literature. 

In the current paper, we discuss the development of nanoparticle-based devices, towards their application as vibration sensors. The devices were developed on top of either flexible Kapton substrates or alumina-modified Kapton substrates. The nanoparticle layer of the devices was developed using the DC magnetron sputtering technique; this vacuum fabrication and deposition method offers facile control of nanoparticle density and size, at room temperature. Platinum was selected as the material for the nanoparticle film since, due to its status as a noble metal with good electrical properties and, most importantly, resistance to oxidation, it guarantees the sensor’s trouble-free operation over extended periods of time (i.e., charge transport unhindered by any oxide layer on the surface of the NPs). The main scope of this work is to present a systematic study of the dynamic behavior of vacuum-grown metallic NP-based resistive strain sensors, at a range of vibration frequencies extending from 20 Hz up to 6250 kHz. To this end, we investigated NP-based strain sensors, which had been found to show increased sensitivity particularly when the NPs are formed on top of a thin alumina film which has been previously deposited on a flexible substrate [25]. Cracks, deliberately created on this insulating layer, play a critical role in current transport through the NP network, rendering this sensor configuration much more sensitive to strain. Taking advantage of this finding, we investigate how the strain sensor sensitivity as well as the NP areal density are related to its response in dynamical excitations. This investigation enables us to shed light on underlying conduction mechanisms and demonstrate at the same time the potential of this device as a low-cost and highly sensitive vibration sensor. The main advantages of the NP sensors discussed herein are the simplicity of their fabrication process as well as their low cost. In addition, their facile integration with flexible substrates due to sensor fabrication at low temperatures renders their use in applications such as e-skin, wearables, etc., attractive.

## 2. Materials and Methods

**Figure 2:**
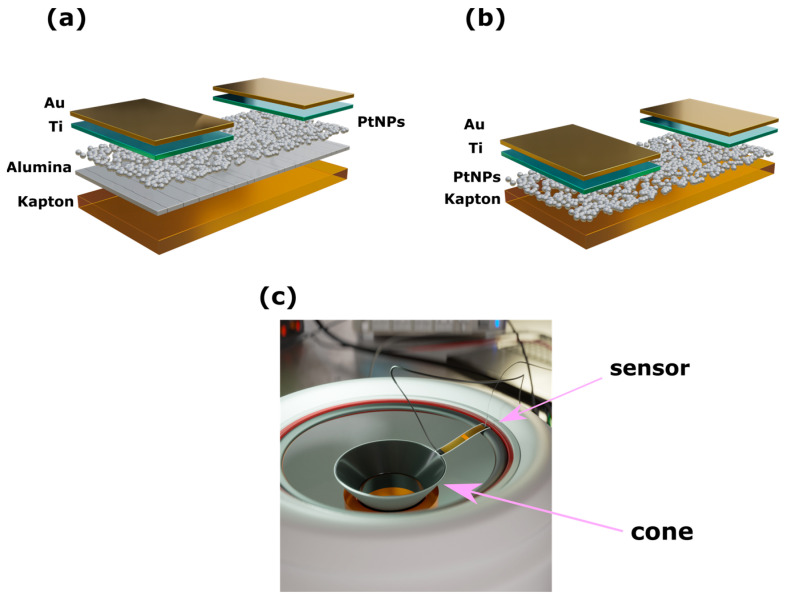
(**a**) Graphical representation of the sensors with the cracked ALD alumina film. (**b**) Graphical representation of the sensors without the cracked alumina film. (**c**) Graphical representation of the home-made experimental setup.

**Figure 3:**
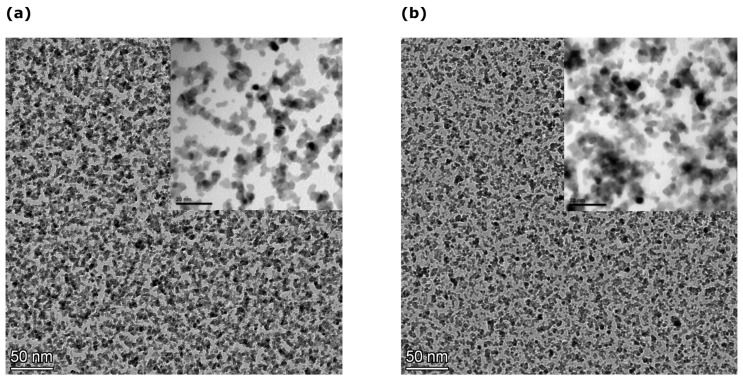
(**a**) TEM image of Pt NPs with surface coverage of 73%. Insert: detailed TEM image. (**b**) TEM image of Pt NPs with surface coverage of 49%. Insert: detailed TEM image.

All sensors were fabricated on top of 120 μm thick Kapton substrates (Goodfellow (Hamburg, Germany) Kapton^®^ HN Film, 0.125 mm thick, 610 mm coil W). In the case of crack-based sensors, a 30 nm thick Al_2_O_3_ (alumina) film was deposited at 150 °C via atomic layer deposition (ALD) on top of the flexible Kapton substrate. Typically, in ALD processing, the overall thickness of the deposited film is controlled via the number of ALD deposition steps or ALD cycles. ALD deposition was conducted using a Picosun ALD R-200 (Espoo, Finland) reactor and under a pressure of 10 mbar. Precursors for the alumina film were deionized (DI) water and tetramethylaluminum (ΤΜA); TMA was purchased from Strem chemicals and was carried into the reaction chamber using nitrogen (N_2_). Inside the deposition chamber, carrier gas flow was maintained at 300 sccm, while for the TMA and DI-water lines, N_2_ flow was set at 150 and 200 sccm, respectively. Finally, exposure time for all precursors was 0.1 s and the time needed for purging the TMA and DI lines was 10 and 15 s, respectively. Two gold electrodes were then patterned via a shadow mask, forming a 150 μm electrode gap; it is worth noting that 150 μm was the minimum feature size that could be reached during the development of the mask. The electrodes were fabricated as follows by using an electron gun evaporator: as a first step, a 4 nm thick titanium (Ti) film was deposited, acting as an adhesion layer; the second and final step of the process was the deposition of 40 nm thick Au film, as can be seen in Figure 2. The lateral dimensions of the finalized electrodes were 2.5 mm × 4 mm. Both electrode and Pt NP depositions were performed at 10**^−^**^6^ mbar pressure. Following the electrodes’ fabrication, Pt nanoparticles (Pt NPs) with an average size of 4 nm were deposited using a modified DC magnetron sputtering system that allowed good control of NP size (±1.5 nm) as well as of surface coverage. It is worth noting that the DC magnetron sputtering technique allows the simultaneous fabrication and deposition of NPs in one single processing step and at room temperature. In the context of the work discussed herein, two different Pt NPs’ surface-coverage densities were used. The first one was characterized by a large NP surface coverage (i.e., 73%, as depicted in Figure 3a), which resulted in an almost closed film of Pt NPs that featured an overall sensor resistance of around 100 Ohms. The second one had an NP surface coverage close to 49% (Figure 3b), falling right below the percolation threshold of the nanoparticle network. This results in devices that feature non-continuous conductive paths within the Pt NP film due to the formation of gaps between distinctive Pt NPs or NP clusters; in this scenario, the overall resistance of the sensors results in values in the order of hundreds of kOhms. For the results discussed in the current paper, four distinctive groups of sensors were developed. The first distinction lies in the usage of different deposition substrates; this results in two sensor groups: in the case of the first group, the sensors were fabricated directly on top of Kapton substrates, while in the case of the second group, the sensors were fabricated on top of a Kapton substrate that was previously modified with a 30 nm thick alumina layer. Each of the above groups (plain Kapton and alumina-modified Kapton) was further separated in two additional sub-groups where two distinctive grades of NP surface coverage (or densities) were used, namely a “high” Pt NP surface coverage and a “low” NP surface coverage. It is also worth remarking that five individual sensors were fabricated in the cases of each of the four sensor groups.

The experimental setup consisted of an HP 8116A function generator, a Teledyne LeCroy WaveAce 2034 (Chestnut Ridge, NY, USA) oscilloscope, a Keithley 6220 (Beaverton, OR, USA) precision current generator, and a 3 inch loudspeaker that was modified in order to expose its central cone. The sensors were fixed at the edge of the immobile loudspeaker chassis and at the mobile central cone of the speaker (see Figure 2c). The speaker was connected to the function generator which drove the speaker cone, forcing it to mechanically oscillate at the input frequency. The current generator was connected to the electrodes of the sensors to apply a constant current while the oscilloscope was also connected to the electrodes. As the sensors were mechanically oscillating due to their connection with the moving loudspeaker, their resistance changed. We used the oscilloscope to monitor the resulting changes in voltage amplitude across the sensor. Due to the fact that not all sensors exhibited the same resistance value, the constant current was not always exactly the same. However, it was adequately modified in order to obtain the same voltage value for all of the sensors that were used throughout our experiments; this value was 10 volts for all sensor groups before applying the mechanical stimulus. 

## 3. Results and Discussion

**Figure 4:**
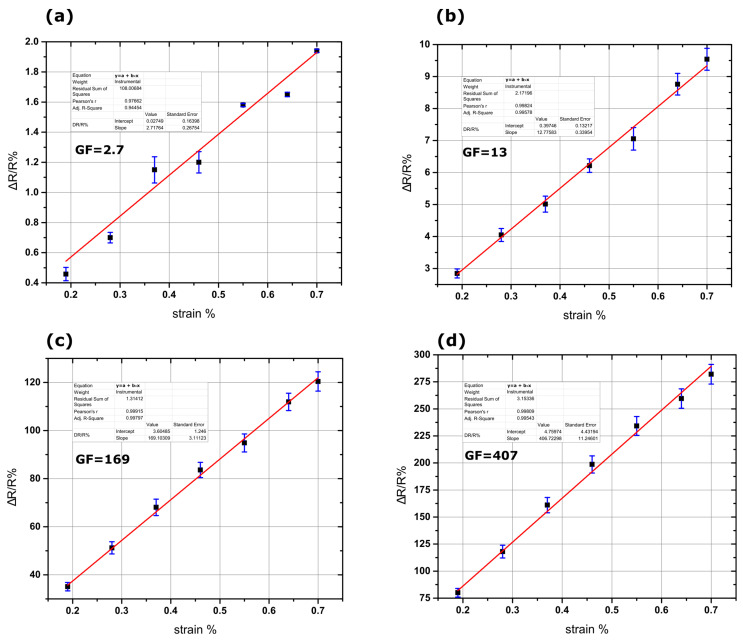
Examples of the calibration curves of the four sensor groups. (**a**) Sensor with dense Pt NPs without alumina showing a GF of 2.7, (**b**) sensor with sparse Pt NPs without alumina showing a GF of 13, (**c**) sensor with dense Pt NPs with 30 nm alumina showing a GF of 169, and (**d**) sensor with sparse Pt NPs with 30 nm alumina showing a GF of 407. All calibration graphs show great linearity with R^2^ coefficient above 0.9.

A first set of experiments was conducted to estimate the sensors’ sensitivity (gauge factor or GF) to strain. For this purpose, the sensors were placed on a home-made stage where the applied strain could be precisely controlled. The maximum applied strain was 0.8%. During the strain application, the resistance was continuously monitored using a Keithley 2400 (Beaverton, OR, USA) multimeter (Figure 4). 

The sensitivities of the strain sensors were calculated measuring the relative resistance change over the applied strain range, by making use of the equation ΔR/R% = GFε%, where ΔR/R% is the relative resistance change, GF the sensitivity, and ε% the strain%. Figure 4 shows the mean value of the sensitivity for each of the four sensor groups, while the error bars represent the standard deviation. All sensors showed great linearity with an R^2^ linear coefficient above 0.9. The alumina-based sensors have very large error bars in comparison with the reference sensors (without the cracked alumina substrate), which we attribute to the random nature of the developed cracks. This is attributed to the nature of the alumina cracks and their random asperity, which resulted in large standard deviation. In addition, the standard deviation of the sensors made on cracked alumina substrate with dense PtNPs (AD PtNPs) and that of sensors made on cracked alumina substrate with sparse PtNPs (AS PtNPs) differ due to the number of conductive paths that were formed within the PtNP networks. However, the presence of the cracks greatly increased the overall sensitivities of the sensors over a large strain range extending from 0,1% up to 7%, as discussed in a previous publication by this group [25]. 

**Figure 5:**
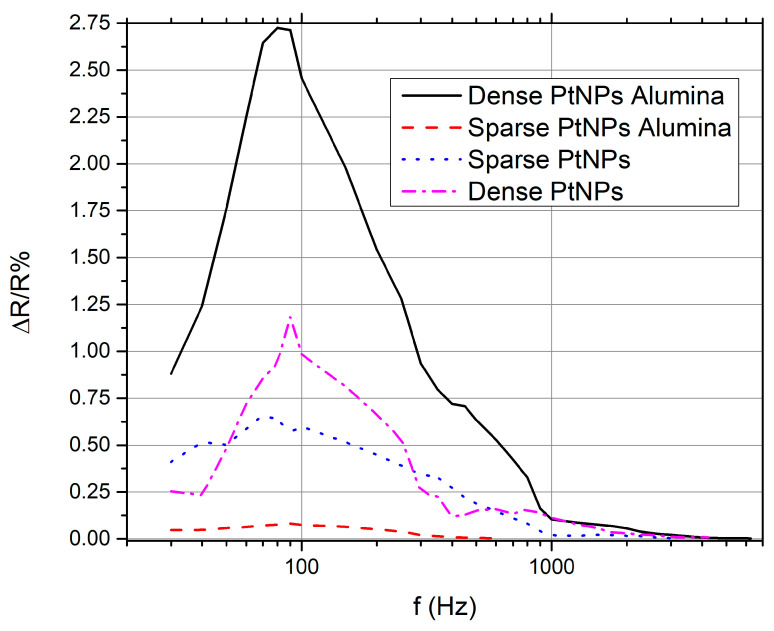
Frequency responses of the four sensor groups: AD (dense PtNPs on alumina), AS (sparse PtNPs on alumina), D (dense PtNPs on polyimide), and S (sparse PtNPs on polymide).

The next set of experiments was designed to investigate the sensors’ performance for vibrations that extend over a wide frequency range. The experiments were carried out using the modified loudspeaker cone which was described in Section 2. In Figure 5, the frequency responses of the four sensor groups can be seen. Frequency response was initially recorded as a function of output voltage; by dividing the output voltage with the fixed current value the voltage modulation was transformed to resistance changes, as seen in Figure 5. The oscilloscope measurement signal was post-processed using fast Fourier transform (FFT) in OriginPro 9 software (rectangle window function). 

The curves in Figure 5 represent the mean values of the response for all measured sensors, per sensor group. Below, we discuss the details for the calculation of the sensors’ response: the sensors’ output was a time-dependent voltage signal that was recorded using the oscilloscope. During the vibration experiments, the sensors were submitted to compressive and tensile strain which resulted in changes in their resistance. Bearing in mind that the sensors were always under a fixed current during the vibration experiments, changes in sensor resistance modulated the output voltage that was recorded using the oscilloscope. By applying the Fourier transformation on the voltage signal and considering the amplitude of the Fourier coefficients as the sensors’ response, we obtained the voltage versus frequency graph. Using the voltage vs frequency dataset, we then obtained Figure 5, where the normalized resistance response (%) of the sensors is shown. The resistance response was calculated by dividing the voltage amplitudes of the Fourier transformation signal by the as measured-fixed value of the current that flowed through each sensor. It is worth noting that the comparison between the different sensor groups was performed using the same parameters for the FFT analysis. Also, all the results are in agreement with the experiments; for example, the measured frequencies were the same as the frequencies that were applied to the speaker.

The sensors on cracked alumina substrate with dense PtNPs (AD PtNPs) exhibited the highest response for all frequencies and the widest frequency response range. However, they do not represent the group with the larger strain sensitivity. Another interesting fact is that the group with the highest strain sensitivity (AS PtNPs) showed a very small frequency response and a quite limited frequency range. Sensor groups without the alumina layer showed similar sensing behavior; to be more specific, the sensor group with the lowest strain sensitivity/performance showed a wider frequency response range than the best-performing sensor group. We remark that the frequency working ranges of the alumina-free sensors were identical. Finally, it is worth noting that almost all sensor groups showed a peak response between 70 Hz and 90 Hz.

**Figure 6:**
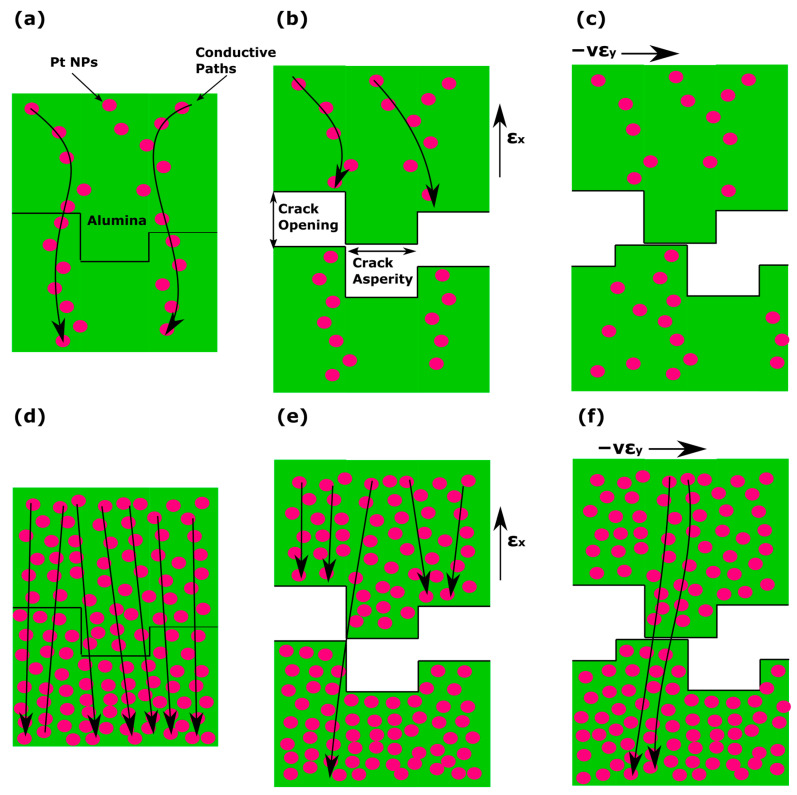
Graphical representation of the crack opening during the measurements. Figures (**a**–**c**) represent the case of sparse Pt NPs. (**a**) Conductive paths (black arrows) through the Pt NPs (pink circles), (**b**) the crack opens with the application of strain, revealing its asperity. The conductive paths are terminated. (**c**) The crack moves parallel to itself due to the Poisson ratio, preventing the reconnection of the conductive paths. Figures (**d**–**f**) represent the dense Pt NPs. (**d**) Conductive paths through the Pt NPs. (**e**) The crack opens with the application of strain, revealing its asperity. Some of the conductive paths are terminated. (**f**) The crack moves parallel to itself due to the Poisson ratio. New conductive paths are formed.

As described herein as well as in previous publications [13,24,25], it is well established that nanoparticle based devices with appropriate nanoparticle density (just below the percolation threshold) feature non-continuous conductive paths within the Pt NP film due to the formation of gaps between distinctive Pt NPs or NP clusters; in this scenario, the overall resistance of the sensors results in values in the order of hundreds of kOhms while their conductivity is dependent on quantum mechanical phenomena such as tunneling and variable range hopping. It is worth noting that in this case, charge transport is extremely sensitive to any inter-particle distance changes (exponentially dependent). At this point it is also worth recalling that, as discussed in detail in [25], the incorporation of an alumina layer in sensor design results in significant improvement in sensor performance and, hence, sensitivity, which in that case was also directly recorded as a relative change in resistance (ΔR/R %). The enhanced sensing performance was due to the formation of cracks in the alumina layer and the resulting increased inter-particle distance compared with alumina-free sensors that were submitted to the same strain. It is, then, evident that the incorporation of an alumina layer results in increased inter-particle distance compared with an alumina-free device for the same amount of strain: the cracks that are inherent in the alumina layer lead to an increased inter-nanoparticle distance for the same applied strain. In the case of vibration sensors made on top of cracked alumina substrates, the experimental findings discussed in the current paper could be explained by taking into account the crack asperity [26] and the Poisson ratio of the substrate [27]. The graphical representation in Figure 6 shows the crack opening of the alumina film when strain was applied. In the case of the AS Pt NPs sensors, when no strain is applied, the current flowing through the Pt NPs (black arrows in Figure 6a) is tuned by the tunneling effect that presents an exponential dependence over interparticle distance. However, since the Pt NPs are sparse, there is only a limited number of conductive paths within the NP network. When strain is applied, the cracks become wider and the number of conductive paths becomes even more limited (Figure 6b). At the same time, the cracks move in a parallel direction relative to the direction of the opening due to the substrate Poisson ratio (Figure 6c). In the case of static measurements, the Kapton substrate returns to its zero-strain position and, as a result, the NP network passes from a high-connectivity state (low resistance) to a low-connectivity state (high resistance) and vice versa, giving rise to a high GF. However, for dynamic measurements, the material cannot return to its zero-strain position; this leads to a permanent displacement and ultimately breaking of some of the original conductive paths, which are no longer capable reconnecting to each other, thus resulting in very small oscillation amplitude of the voltage. In the case of the AD Pt NPs sensors, the conductive paths are numerous (Figure 6d). When strain is applied, some of them no longer contribute to electrical conduction, since the wider gaps prevent tunneling and, thus, transport along these paths is blocked (Figure 6e). However, during the dislocation of the cracks, the probability of creating new conductive paths is high due to the high density of the Pt NPs. This results in significant voltage oscillation amplitude over a wide frequency range. As noted above, the trend shown in Figure 5 is similar for alumina-free sensors; sparse NP sensors were less sensitive than dense NP sensors, in contrast with static strain measurements shown in Figure 4. However, it should also be underlined that the differences between these two sensor groups were much smaller than in the case of cracked alumina substrate sensors and, interestingly, for frequencies below 40 Hz there was a reversal of this trend. These relatively small differences between the two alumina-free sensor groups can be attributed mainly to the inability of Kapton substrate to return to its initial no-strain position after being subjected to higher oscillation frequencies. For alumina-free sparse NP networks, this effect again leads to a permanent disruption of the inter-NP conductive paths that again cannot be replaced by the formation of new charge transport paths, hence resulting in smaller changes of the oscillating voltage; at the same time and for dense NP networks, this effect is overcome by the large number of conductive paths. It is also worth noting that one potential drawback that could be associated with the use of an alumina layer is its potential deterioration under repetitive vibration experiments. However, during our vibration experiments, the alumina-based sensors were submitted to vibrations/oscillations over a wide range of frequencies; this translates to a vast number of compressive and tensile strain cycles while the sensors were oscillating.

The experimental observations discussed above could also be attributed to the detachment of nanoparticles from their positions during the Kapton mechanical oscillations, a hypothesis that is difficult to validate experimentally. This explanation appears, however, quite unlikely since in such a case, we would expect large changes in the as-measured resistance. This argument in particular is stronger in the case of sensors with dense nanoparticles where just after the NP deposition experiments, we did not observe any significant difference of resistance values from sample to sample. We cannot, however, completely exclude that both phenomena, the one illustrated in Figure 6 and the one related with nanoparticle detachment, were present. 

**Figure 7:**
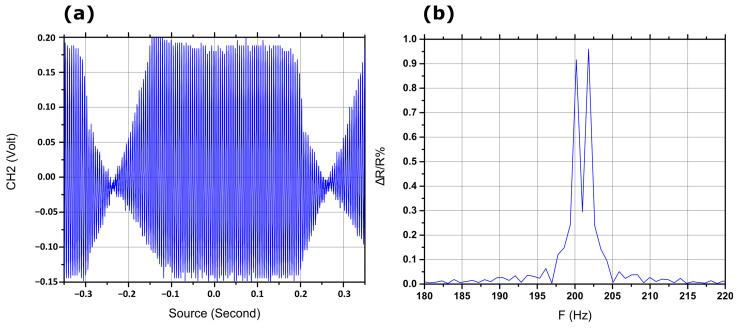
Sensor response at two neighboring frequencies, 200 Hz and 202 Hz. (**a**) Raw signal from the oscilloscope, (**b**) ΔR/R% after the fast Fourier transformation.

Finally, a very interesting property of the sensors, namely their resolution ability for neighboring frequencies, was examined. For this purpose, all sensors were subjected to simultaneous oscillation at neighboring frequencies i.e., 200 Hz and 202 Hz, while the voltage modulation was monitored and recorded via the oscilloscope. As can be seen in Figure 7, the sensors can easily distinguish between neighboring frequencies following FFT processing of the measured signal in the time domain. 

## 4. Conclusions

In this work, we investigated the mechanical response of a nanoparticle-based resistive strain sensor, to forced vibrations and as a function of excitation frequency. The vibration sensors that are reported in the current article were fabricated on a flexible Kapton substrate that was either unmodified or covered with a thin and cracked alumina layer. Naked or chemically unmodified Pt nanoparticles were produced via the DC magnetron sputtering technique, in a single fabrication step (simultaneous fabrication and deposition) and at room temperature. The alumina layer was produced at 150 °C via the atomic layer deposition technique; it is worth noting that in the case of alumina functionalized substrates, after repetitive bending cycles and due to the mechanical properties of the alumina layer, cracks formed prior to any nanoparticle deposition. It was shown that nanoparticle surface density (controlled via the overall deposition time during their deposition in a vacuum) plays an important role in determining the sensitivity of the sensor response for excitations within the frequency range reported herein. At the same time, nanoparticle density is not the only critical factor that determines sensor sensitivity; to be more specific, the employment of a cracked alumina layer also contributed to increased sensor performance. Interestingly, we report here that the highest sensitivity was found for a dense nanoparticle network; this type of behavior is opposite to what has been observed in the past for the same sensors (nanoparticle sensors on cracked alumina substrates) for static (i.e., vibration-free) strain measurements. To explain these findings, we suggest a physical mechanism of current transport via tunneling, which, being sensitive to interparticle distance, is modified during vibrations in a different manner depending on the nanoparticle density and substrate surface. 

The sensors were submitted to a wide range of controlled vibrations at various frequencies and exhibited good response from relatively low frequencies up to 400 Hz, while they can detect high frequency mechanical signals up to 6250 Hz. Moreover, the sensors showed remarkable signal frequency detection resolution, which allowed them to detect neighboring frequencies with resolution as low as 2 Hz. The best results were obtained for a high nanoparticle surface density that was substantially above the percolation threshold. In all, the vibration sensors that are discussed in this paper and are based on advanced materials offer increased sensitivity compared with traditional competing technologies and show great promise towards future integration in acoustics, voice-recognition, soft robotics, e-skin, etc. One of the main challenges to their wider use remaining to be investigated is their robustness, which depends on the number of operation cycles (endurance) for which the mechanical properties of the cracked alumina layer remain stable. In addition, the use of a Wheatstone bridge configuration for sensor measurement could be investigated to integrate the sensor in autonomous systems.

## Data Availability

Dataset available on request from the authors.
